# Reply to: intensive care unit-to-unit capacity transfers are associated with increased mortality—no hasty conclusions in the event of a crisis

**DOI:** 10.1186/s13613-022-01032-6

**Published:** 2022-07-09

**Authors:** Fredric Parenmark, Sten M. Walther

**Affiliations:** 1 Centre for Research and Development, Uppsala University, Region Gävleborg, Gävle, Sweden; 2 Department of Anaesthesia and Intensive Care, Gävle Hospital, Gävle, Sweden; 3 Department of Medical and Health Sciences, Faculty of Health Sciences, Linköping University, Linköping, Sweden; 4 Present Address: Department of Cardiothoracic Anaesthesia and Intensive Care, Linköping University Hospital, Linköping, Sweden

We thank Painvin and co-writers for their kind and relevant comment regarding our study, in which we compared three types of ICU-to-ICU transfers in regards to 30 day mortality [1].

As pointed out in their comment, there could be several factors contributing to the results. They particularly mentioned night-time transfers and lack of ICU-beds. While discharge from ICU at night increases risk of death, it is worth noting that in our analysis, the increased risk associated with transfer remained also after adjusting for night-time transfer. This indicates that other explanations, in addition to transfer at night, must be sought.

We examined 3 years preceding the pandemic and agree that our results cannot be extrapolated to circumstances during the pandemic. In fact, roughly one quarter of patients admitted with COVID-19 to Swedish ICUs underwent at least one ICU-to-ICU transfer during 2020–2021. Very few of these were for intensive treatment at a higher care level. While the effect on ultimate outcome of this high transfer rate remains to be determined, a sudden increase of local ICU-bed availability to handle surges in patient flow may not be the best solution [2].

The increased risk associated with capacity transfer compared to repatriation or clinical transfer is of great concern. In addition, as mentioned by Painvin et al., there could be ways to reduce this by increasing the number of beds and trained staff in the ICU as well as promote structured handovers and avoid ad hoc transport methods. What the best solutions really are is yet to be seen and we agree that further studies on the different aspects of critical care transfers are needed. Particularly, whether and in what way capacity transfer effects outcome, which, as also stated in the letter, involves identifying the appropriate control patient for comparisons of outcome.

Fredric Parenmark, Sten M Walther.

## Data Availability

Not applicable.
